# Synthesis, Antimicrobial, and Anti-Proliferative Activities of Novel 4-(Adamantan-1-yl)-1-arylidene-3-thiosemicarbazides, 4-Arylmethyl *N*′-(Adamantan-1-yl)piperidine-1-carbothioimidates, and Related Derivatives

**DOI:** 10.3390/molecules24234308

**Published:** 2019-11-26

**Authors:** Aamal A. Al-Mutairi, Monirah A. Al-Alshaikh, Fatmah A. M. Al-Omary, Hanan M. Hassan, Areej M. El-Mahdy, Ali A. El-Emam

**Affiliations:** 1 Department of Chemistry, College of Sciences, Al Imam Mohammad Ibn Saud Islamic University (IMSIU), Riyadh 11623, Saudi Arabia; aamutairi@imamu.edu.sa; 2 Department of Chemistry, College of Sciences, King Saud University, Riyadh 11451, Saudi Arabia; mshaikh@ksu.edu.sa; 3 Department of Pharmaceutical Chemistry, College of Pharmacy, King Saud University, Riyadh 11451, Saudi Arabia; Falomary@ksu.edu.sa; 4 Department of Pharmacology and Biochemistry, Department of Pharmacology and Biochemistry, College of Pharmacy, Delta University for Science and Technology, International Costal Road, Gamasa City, Mansoura 11152, Dakahliya, Egypt; hananhafila@hotmail.com; 5 Department of Pharmaceutical Sciences, College of Pharmacy, Princess Nourah bint Abdulrahman University, Riyadh 11671, Saudi Arabia; areej_243@yahoo.com; 6 Department of Microbiology and Immunology, Faculty of Pharmacy, University of Mansoura, Mansoura 35516, Egypt; 7 Department of Medicinal Chemistry, Faculty of Pharmacy, University of Mansoura, Mansoura 35516, Egypt

**Keywords:** adamantane, carbothioimidates, thiazolidin-4-ones, antimicrobial activity, anti-proliferative activity

## Abstract

The reaction of 4-(adamantan-1-yl)-3-thiosemicarbazide **3** with various aromatic aldehydes yielded the corresponding thiosemicarbazones **4a–g**. 1-Adamantyl isothiocyanate **2** was reacted with 1-methylpiperazine or piperidine to yield the corresponding *N*-(adamantan-1-yl)carbothioamides **5** and **6**, respectively. The latter was reacted with benzyl or substituted benzyl bromides to yield the *S*-arylmethyl derivatives **7a–c**. Attempted cyclization of 1,3-bis(adamantan-1-yl)thiourea **8** with chloroacetic acid via prolonged heating to the corresponding thiazolidin-4-one **9** resulted in desulfurization of **8** to yield its urea analogue **10**. The thiazolidin-4-one **9** and its 5-arylidene derivatives **11a,b** were obtained via microwave-assisted synthesis. The in vitro antimicrobial activity of the synthesized compounds was evaluated against a panel of Gram-positive and Gram-negative bacteria and yeast-like pathogenic fungus *Candida albicans*. Compounds **7a–c** displayed marked broad spectrum antibacterial activities (minimal inhibitory concentration (MIC), 0.5–32 μg/mL) and compounds **4a** and **4g** showed good activity against *Candida albicans*. Nine representative compounds were evaluated for anti-proliferative activity towards three human tumor cell lines. Compounds **7a–c** displayed significant generalized anti-proliferative activity against all the tested cell lines with IC_50_ < 10 μM.

## 1. Introduction

**Figure 1:**
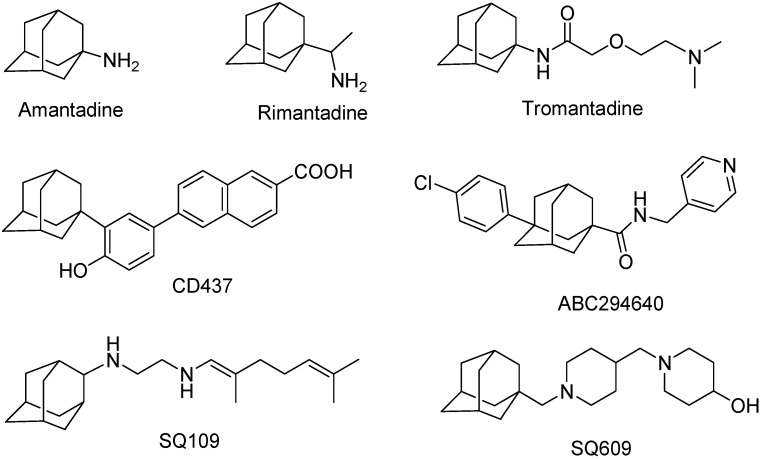
Adamantane-based chemotherapeutic drugs.

As a result of the evolution of new resistant bacterial, fungal, and viral strains, the development of new chemotherapeutic agents is becoming the major priority in pharmaceutical research with the aim to discover newer, more potent molecules with higher specificity and reduced toxicity than the existing ones. Adamantane-based derivatives are currently used as efficient therapies for the treatment of various pathological disorders [1,2,3]. As a result of the high lipophilicity of adamantane, the incorporation of an adamantyl moiety into structure of bioactive compounds positively modulates the biological activity. Amantadine [4,5] and rimantadine [6] were early approved as potent therapy against Influenza A viral infections, and tromantadine is currently used against herpes simplex skin viral infections [7]. In addition, several adamantane-based analogues were proven to possess significant inhibitory activity against human immunodeficiency viruses (HIV) [8,9,10]. Antitumor activity was reported for some adamantane derivatives, the synthetic retinoid CD437 was discovered as a potent inducer of apoptosis in human head and neck squamous cell carcinoma [11,12]. ABC294640 is a recently approved anticancer drug for the treatment of patients with advanced solid tumors [13,14]. Moreover, several adamantane-based derivatives were recognized as potent bactericidal and fungicidal agents [15,16,17]. SQ109 is a newly developed drug for the treatment of tuberculosis (TB); it was approved for use against drug-susceptible and drug-resistant TB strains [18]. The related dipiperidine derivative SQ609 was further discovered as a lead compound with potent long acting activity against *Mycobacterium tuberculosis* [19] (Figure 1).

On the other hand, thiosemicarbazide and thiosemicarbazone derivatives [20,21,22], isothiourea [16,23,24], and 4-thiazolidinone [25,26,27] derivatives were reported to possess marked chemotherapeutic properties.

In view of the above mentioned observations and in continuation to an ongoing study on the chemical and pharmacological properties of adamantane-based derivatives [10,16,17], herein we report the synthesis and characterization of novel adamantane derivatives containing thiosemicarbazide, isothiourea, or 4-thiazolidinone moieties as potential antibacterial, antifungal, and/or anti-proliferative agents.

## 2. Results and Discussion

### 2.1. Chemical Synthesis

**Scheme 1:**
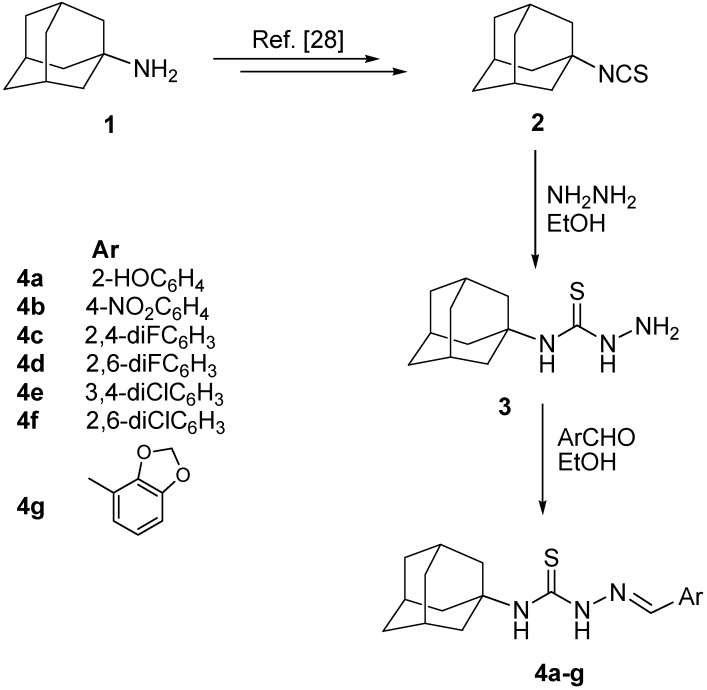
Synthetic approach for the target compounds **4a–g**.

Adamantan-1-yl isothiocyanate **2** was prepared in good yield starting from 1-adamantylamine **1** following our previously described procedure [28] via modification of the general methods of Munch et al. [29] and Spilovska et al. [30]. The intermediate 4-(adamantan-1-yl)-3-thiosemicarbazide **3** was previously reported as a minor byproduct during the reaction of *N*-(adamantan-1-yl)- 4-ethoxycarbonylpiperidine-1-carbothioamide with excess hydrazine hydrate, in ethanol, at reflux temperature [29]. In the present investigation, compound **3** was prepared in high yield (94%) via treatment of adamantan-1-yl isothiocyanate **2** with hydrazine hydrate in ethanol at reflux temperature for one hour. 4-(Adamantan-1-yl)-3-thiosemicarbazide **3** was then condensed with the aromatic aldehydes; 2-hydroxybenzaldehyde, 4-nitrobenzaldehyde, 2,4-difluorobenzaldehyde, 2,6-difluorobenzaldehyde, 3,4-dichlorobenzaldehyde, 2,6-dichlorobenzaldehyde, or benzo[*d*][1,3]dioxole-4-carbaldehyde via heating in ethanol to yield the corresponding azomethine derivatives **4a–g** in 68%–85% yield (Scheme 1). Compound **4a** was previously reported as a potential treatment for neurodegerative diseases such as Alzheimer’s disease [31]. The structures of compounds **4a–g** were confirmed on the basis of ^1^H nuclear magnetic resonance (NMR), ^13^C NMR, and electrospray ionization mass spectral (ESI/MS) data, which showed their negative ion peaks [M − H]^−^.

**Scheme 2:**
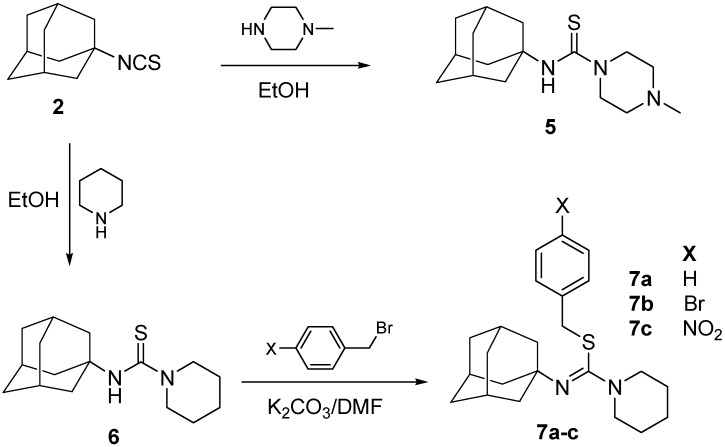
Synthetic approach for the target compounds **5**, **6**, and **7a–c**.

Adamantan-1-yl isothiocyanate **2** was reacted with 1-methylpiperazine or piperidine in boiling ethanol to yield the corresponding *N*-(adamantan-1-yl)carbothioamides **5** and **6**, respectively. Compound **6** was previously reported [28]. The reaction of the carbothioamide derivative **6** with benzyl or 4-substituted benzyl bromides in *N*,*N*-dimethylformamide (DMF) in the presence of anhydrous potassium carbonate at room temperature yielded the corresponding *S*-arylmethyl (isothiourea) derivatives **7a–c** in good yields (Scheme 2). The structures of compounds **5** and **7a–c** were confirmed on the basis of ^1^H and ^13^C NMR spectra, in addition to ESI/MS data, which showed their positive ion peaks [M + H]^+^.

**Figure 2:**
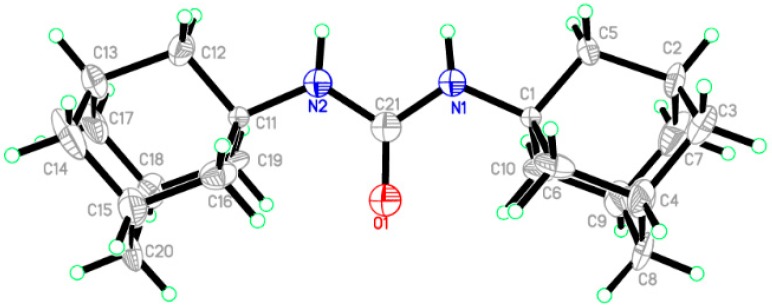
The Oak Ridge Thermal Ellipsoid Plot (ORTEP) plot of compound **10** showing 40% probability displacement ellipsoids for non-H atoms.

The reaction of adamantan-1-yl isothiocyanate **2** with 1-adamantylamine **1** by heating in ethanol under reflux for four hours yielded the symmetric thiourea derivative **8** in 75% yield. The reaction of monosubstituted or 1,3-disubstituted symmetric thiourea derivatives with chloroacetic acid or ethyl chloro- or bromoacetate in the presence of sodium acetate was reported to yield the corresponding iminothiazolidin-4-one derivatives [25,26,27,32]. Attempted reaction of the symmetric thiourea derivative **8** with chloroacetic acid via heating in ethanol in the presence of sodium acetate for up to four hours to get the intermediate 3-(adamantan-1-yl)-2-(adamantan-1- ylimino)thiazolidin-4-one **9** was unsuccessful and the reactants were recovered unchanged. Increasing the reaction time to 10–12 h resulted in desulphurization of the thiourea derivative **9** to yield the corresponding urea analogue **10**. Although this type of desulfurization is uncommon, similar reactions were previously reported [33,34]. The assignment of the structures of compound **10** was based on its physical and spectral data, which were identical to the reported data [35,36]. In addition, the structure was further supported by single crystal X-ray diffraction (Figure 2).

**Scheme 3:**
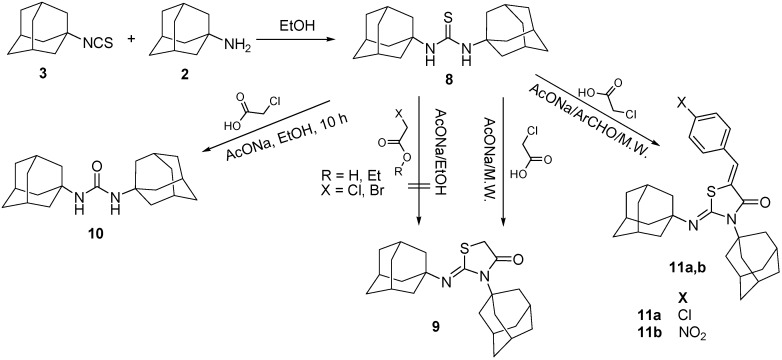
Synthetic approach for the target compounds **8**, **9**, and **11a,b**.

Microwave irradiation was introduced as a useful alternative to traditional heating for the synthesis of several heterocyclic derivatives [37,38,39]. Thus, it was of interest to try this environmentally friendly tool to get thiazolidin-4-one derivative **9**. After several pilot experiments to optimize the irradiation time and intensity, it was found that microwave irradiation for 10 min, a maximum power of 700 W is the optimum condition for this reaction, and the product was attained in 72% yield. The successful microwave assisted synthesis of compound **9** prompted us to try a three-component one step reaction of compound **8** with chloroacetic acid and the appropriate aromatic aldehyde, in ethanol, in the presence of anhydrous sodium acetate. The trial was successful and the products **11a** and **11b** were obtained in fair yields after being irradiated for 10 min at a maximum power of 700 W (Scheme 3).

### 2.2. In Vitro Antimicrobial Activity

The in vitro growth inhibitory activity of the newly synthesized compounds **4a–g**, **5**, **7a–c**, **8**, **9**, **11a**, and **11b** was assessed against the standard bacterial strains of the American type culture collection (ATCC), *Staphylococcus aureus* ATCC 6571, *Bacillus subtilis* ATCC 5256, *Micrococcus luteus* ATCC 27141 (Gram-positive bacteria), *Escherichia coli* ATCC 8726, *Pseudomonas aeruginosa* ATCC 27853 (Gram-negative bacteria), and the yeast-like pathogenic fungus *Candida albicans* MTCC 227. The primary antimicrobial screening was carried out using the semi-quantitative agar-disc diffusion method with Müller–Hinton agar medium [40]. The results of the preliminary antimicrobial testing of compounds **4a–g**, **5**, **7a–c**, **8**, **9**, **11a**, and **11b** (200 μg/disc); the antibacterial antibiotics Gentamicin sulfate, Ampicillin trihydrate, and the antifungal drug Clotrimazole (100 μg/disc); and the calculated log *p*-values (Clog *P*) of the compounds (calculated using the CS ChemOffice Ultra version 8.0, CambridgeSoft, Cambridge, MA, USA) are listed in Table 1.

The results indicated that the tested compounds showed various levels of activity against the tested microorganisms. Potent antibacterial activity was displayed by the compounds **4a**, **4c**, **4d**, **4e**, **4f**, **4g**, **7a**, **7b**, and **7c**, which displayed growth inhibition zones ≥18 mm against one or more of the tested microorganisms. Meanwhile, the compounds **4b** and **5** showed moderate activity (growth inhibition zones 14–17 mm); the compounds **8**, **11a**, and **11b** were poorly active (growth inhibition zones 10–13 mm); and compound **9** was practically inactive (growth inhibition zones ≤ 10 mm) against the tested microorganisms. In general, the tested Gram-positive bacteria are considered the most sensitive among the tested bacterial strains and the activity against the tested Gram-negative bacteria was generally lower than that of the Gram-positive bacteria. Compounds **4a**, **4c**, **4d**, **4e**, **4e**, **4f**, **4g**, **7a**, **7b**, and **7c** displayed potent activity particularly against the Gram-negative bacteria *Escherichia coli* and the optimum antibacterial activity was attained by compounds **4a**, **4d**, **4f**, **7b**, and **7c**, which exhibited potent broad spectrum activity against all the tested bacterial strains. The antifungal activity of the compounds against *Candida albicans* was generally lower than their antibacterial activity, compounds **4a** and **4g** showed potent activity; compound **4f** displayed moderate activity; and compounds **4b**, **4c**, **4f**, **7a**, **7b**, and **7c** displayed marginal activity compared with Clotrimazole.

The minimal inhibitory concentrations (MICs) of the most active compounds **4a**, **4c**, **4d**, **4e**, **4f**, **4g**, **7a**, **7b**, and **7c**, as well as the antibacterial antibiotics Gentamicin sulfate, Ampicillin trihydrate, and the antifungal drug Clotrimazole, were determined using the microdilution susceptibility method in Müller–Hinton broth and Sabouraud liquid medium [41]. The MIC values were almost consistent with the results obtained in the primary screening.

According to the results of the antimicrobial activity, it could be concluded that the 4-(adamantan-1-yl)-1-arylidene-3-thiosemicarbazides **4a–g** and the 4-arylmethyl *N*′-(adamantan-1-yl)piperidine-1-carbothioimidates **7a–c** are the most active derivatives. Among the 4-(adamantan-1-yl)-1-arylidene-3-thiosemicarbazide series **4a–g**, it was observed that the hydroxy- and benzodioxole substituents (**4a** and **4g**) are optimal for antifungal activity. The 2,6-dihalophenyl analogues **4d** and **4f** showed higher potency against the tested Gram-negative bacteria compared with their 2,4- or 3,4-dihalophenyl analogues **4c** and **4e**, which showed potent activity against the tested Gram-positive bacteria. In addition, the antibacterial activity of compounds **4a–g** was found to be correlated to their lipophilicty, in contrast to their antifungal activity. The thiourea derivatives **5** and **8** showed moderate and weak activity against the tested Gram-positive bacteria. The insertion of the isothiourea moiety (compounds **7a–c**) greatly enhanced the antibacterial potency and spectrum as the piperidine-4-carbothioimidates **7a–c** displayed marked broad spectrum antibacterial activity with marginal antifungal activity. On the other hand, the conversion of the thiourea derivative **8** to its corresponding thiazolidine analogue **9** resulted in a total loss of antibacterial activity. The insertion of a 4-arylidene moiety on the thiazolidine derivative **9**, which resulted in the more lipophilic derivatives **11a** and **11b**, resulted in limited improvement in the activity against the tested Gram-positive bacteria.

### 2.3. In Vitro Anti-Proliferative Activity

The in vitro anti-proliferative activity of nine representative compounds (**4a**, **4d**, **4f**, **4g**, **7a**, **7b**, **7c**, **9**, and **11a**) was assessed against three human tumor cell lines; namely, HL-60 (human promyelocytic leukemia cell line), HT-29 (human colorectal cancer cell line), and MCF7 (human breast cancer cell line) using the 3-[4,5-dimethylthiazoyl-2-yl]-2,5-diphenyltetrazolium bromide (MTT) assay [42,43,44]. Compounds **4a**, **4d**, **4f**, **4g**, **7a**, **7b**, **7c**, **9**, and **11a** were selected after preliminary pilot experiments, which proved that the other derivatives were almost inactive (IC_50_ > 100 μM). The results of the anti-proliferative activity of compounds **4a**, **4d**, **4f**, **4g**, **7a**, **7b**, **7c**, **9**, **11a**, and the potent anticancer drug Doxorubicin [45] are shown in Table 2.

The results indicated that the tested compounds displayed variable degrees of anti-proliferative activity against the tested cancer cell lines. The optimal activity was attained by the isothiourea derivatives **7a**, **7b**, and **7c** with IC_50_ <10 μM against the tested cell lines. Meanwhile, compounds **4d**, **4f**, and **4g** showed moderate activity with IC_50_ values>10–50 μM; compound **4a** exhibited marginal activity against HT-29 and MCF7 cell lines. In addition, the thiazolidinone derivatives **9** and **11a** did not show any activity on the tested cell lines (IC_50_ > 100 μM).

According to the results of the anti-proliferative activity, it could be concluded that the arylmethyl *N*′-(adamantan-1-yl)piperidine-1-carbothioimidates **7a–c** and, to a lesser extent, the 4-(adamantan-1-yl)-1-arylidene-3-thiosemicarbazides **4a,d,f,g**, are the most active derivatives. In the adamantyl piperidine-4-thiocarboxamide series **7a–c**, it seems that the conjugation of the adamantyl moiety with an isothiourea fragment is optimal for anti-proliferative activity, regardless of the nature of the arylmethyl substituents (X). In addition, the benzodioxole substituent enhanced the anti-proliferative activity of the 4-(adamantan-1-yl)-1-arylidene-3-thiosemicarbazide.

## 3. Materials and Methods

### 3.1. General Information

Melting points (°C) were measured in open glass capillaries using IA9100 electrothermal melting point apparatus and are uncorrected. Nuclear magnetic resonance (NMR) spectra were obtained on a Bruker AC 500 Ultra Shield NMR spectrometer at 500.13 MHz for ^1^H and 125.76 MHz for ^13^C and Bruker Ascend 700 NMR spectrometer at 700.17 MHz for ^1^H and 176.08 MHz for ^13^C; the chemical shifts are expressed in δ (ppm) downfield from tetramethylsilane (TMS) as internal standard; coupling constants *(J*) are expressed in Hz. Deuteriochloroform (CDCl_3_) and deuteriodimethyl sulfoxide (DMSO-*d*_6_) were used as solvents. Electrospray ionization mass spectra (ESI-MS) were recorded on an Agilent 6410 Triple Quad tandem mass spectrometer (Agilent Technologies, Santa Clara, CA, USA) at 4.0 kV for positive ions. Microwave irradiation was carried on using a domestic microwave oven (Haas HMW 20WN) at 700 W. Elemental analyses (C, H, N, and S) were in agreement with the proposed structures within ±0.4% of the theoretical values (Table S1). Monitoring of the reactions and checking of the purity of the final products were carried out by thin layer chromatography (TLC) using silica gel precoated aluminum sheets (60 F_254_; Merck Schuchardt, Darmstadt, Germany), as well as visualization with ultraviolet light (UV) at 365 and 254 nm. The reference drugs Gentamicin sulfate (CAS 1405-41-0), Ampicillin trihydrate (CAS 7177-48-2), Clotrimazole (CAS 23593-75-1), and Doxorubicin (CAS 23214-92-8) were purchased from Sigma-Aldrich Chemie GmbH, Germany. The microanalytical data (C, H, N and S) and the experimental details of the determination of in vitro antimicrobial activity, in vitro anti-proliferative activity are given in supplementary materials.

### 3.2. 4-(Adamantan-1-yl)-3-thiosemicarbazide ***3***

Hydrazine hydrate (98%, 5 mL) was added to a hot solution of 1-adamantyl isothiocyanate **2** (1.93 g, 0.01 mol) in ethanol (10 mL) and the mixture was heated under reflux with stirring for one hour. On cooling, the precipitated crude product was filtered, washed with cold ethanol, dried, and crystallized from ethanol to yield 2.12 g (94%) of the target compound **3** as fine colorless needle crystals; m.p. 195–197 °C [29].

### 3.3. 4-(Adamantan-1-yl)-1-arylidene-3-thiosemicarbazides ***4a–g***

A mixture of 4-(adamantan-1-yl)-3-thiosemicarbazide **3** (0.45 g, 2.0 mmol) and the appropriate aromatic aldehyde (2.0 mmol), in ethanol (10 mL), was heated under reflux with stirring for four hours. On cooling, the precipitated crude products were filtered, washed with cold ethanol, dried, and crystallized from ethanol (**4a**, **4c**, **4d**, and **4g**) or ethanol/chloroform (**4b**, **4e**, and **4f**) as transparent needle or block crystals.

*4-(Adamantan-1-yl)-1-(2-hydroxybenzylidene)-3-thiosemicarbazide***4a**: Yield 70%; m.p. 194–196 °C (EtOH); Mol. Formula (Mol. Wt.): C_18_H_23_N_3_OS (329.46). ^1^H NMR (DMSO-*d*_6_, 700.17 MHz): δ 1.66 (s, 6H, Adamantane-H), 2.08 (s, 3H, Adamantane-H), 2.27 (s, 6H, Adamantane-H), 6.84–6.89 (m, 2H, Ar-H), 7.23 (t, 1H, Ar-H, *J* = 7.0 Hz), 7.48 (s, 1H, NH), 7.68-7.71 (m, 1H, Ar-H), 8.38 (s, 1H, CH=N), 10.0 (br. s, 1H, OH), 11.30 (s, 1H, NH). ^13^C NMR (DMSO-*d*_6_, 176.08 MHz): δ 29.48, 36.39, 40.43, 53.40 (Adamantane-C), 116.58, 119.86, 120.76, 126.0, 131.61, 157.61 (Ar-C), 139.01 (CH=N), 175.30 (C=S). ESI-MS, *m*/*z*: 328.3 [M − H]^−^.

*4-(Adamantan-1-yl)-1-(4-nitrobenzylidene)-3-thiosemicarbazide***4b**: Yield 85%; m.p. 153–155 °C (EtOH/CHCl_3_); Mol. Formula (Mol. Wt.): C_18_H_22_N_4_O_2_S (358.46). ^1^H NMR (DMSO-*d*_6_, 700.17 MHz): δ 1.66 (s, 6H, Adamantane-H), 2.09 (s, 3H, Adamantane-H), 2.30 (s, 6H, Adamantane-H), 7.58 (s, 1H, NH), 7.94 (d, 2H, Ar-H, *J* = 7.0 Hz), 8.24 (d, 2H, Ar-H, *J* = 7.0 Hz), 8.15 (s, 1H, CH=N), 11.64 (s, 1H, NH). ^13^C NMR (DMSO-*d*_6_, 176.08 MHz): δ 29.47, 36.37, 40.21, 53.80 (Adamantane-C), 124.45, 128.37, 140.82, 148.09 (Ar-C), 139.24 (CH=N), 175.27 (C=S). ESI-MS, *m*/*z*: 357.3 [M − H]^−^.

*4-(Adamantan-1-yl)-1-(2,4-difluorobenzylidene)-3-thiosemicarbazide***4c**: Yield 72%; m.p. 216–218 °C (EtOH); Mol. Formula (Mol. Wt.): C_18_H_21_F_2_N_3_S (349.44). ^1^H NMR (DMSO-*d*_6_, 700.17 MHz): δ 1.65 (s, 6H, Adamantane-H), 2.08 (s, 3H, Adamantane-H), 2.28 (s, 6H, Adamantane-H), 7.15–7.17 (m, 1H, Ar-H), 7.32–7.35 (m, 2H, Ar-H), 7.51 (s, 1H, NH), 7.79–8.0 (m, 1H, Ar-H), 8.22 (s, 1H, CH=N), 11.47 (s, 1H, NH). ^13^C NMR (DMSO-*d*_6_, 176.08 MHz): δ 29.47, 36.37, 40.32, 53.60 (Adamantane-C), 104.80, 112.91, 118.90, 128.71, 160.59, 162.03 (Ar-C), 133.99 (CH=N), 175.11 (C=S). ESI-MS, *m*/*z*: 348.4 [M − H]^−^.

*4-(Adamantan-1-yl)-1-(2,6-difluorobenzylidene)-3-thiosemicarbazide***4d**: Yield 68%; m.p. 193–195 °C (EtOH); Mol. Formula (Mol. Wt.): C_18_H_21_F_2_N_3_S (349.44). ^1^H NMR (DMSO-*d*_6_, 700.17 MHz): δ 1.66 (s, 6H, Adamantane-H), 2.08 (s, 3H, Adamantane-H), 2.23 (s, 6H, Adamantane-H), 7.18–7.21 (m, 2H, Ar-H), 7.49–7.53 (m, 2H, Ar-H & NH), 8.20 (s, 1H, CH=N), 11.59 (s, 1H, NH). ^13^C NMR (DMSO-*d*_6_, 176.08 MHz): δ 29.41, 36.29, 41.33, 53.35 (Adamantane-C), 111.81, 112.93, 131.98, 160.4 (Ar-C), 132.04 (CH=N), 175.14 (C=S). ESI-MS, *m*/*z*: 348.3 [M − H]^−^.

*4-(Adamantan-1-yl)-1-(3,4-dichlorobenzylidene)-3-thiosemicarbazide***4e**: Yield 78%; m.p. 226–228 °C (EtOH/CHCl_3_); Mol. Formula (Mol. Wt.): C_18_H_21_Cl_2_N_3_S (382.35). ^1^H NMR (DMSO-*d*_6_, 700.17 MHz): δ 1.66 (s, 6H, Adamantane-H), 2.08 (s, 3H, Adamantane-H), 2.23 (s, 6H, Adamantane-H), 7.53 (s, 1H, NH), 7.67–7.86 (m, 2H, Ar-H), 7.97 (d, 1H, Ar-H, *J* = 7.0 Hz), 8.03 (s, 1H, CH=N), 11.49 (s, 1H, NH). ^13^C NMR (DMSO-*d*_6_, 176.08 MHz): δ 29.47, 36.38, 40.32, 53.73 Adamantane-C), 127.35, 129.03, 131.44, 132.20, 135.28, 132.49 (Ar-C), 139.24 (CH=N), 175.15 (C=S). ESI-MS, *m*/*z*: 380.4 [M − H]^−^, 382.4 [M + 2 − H]^−^.

*4-(Adamantan-1-yl)-1-(2,6-dichlorobenzylidene)-3-thiosemicarbazide***4f**: Yield 75%; m.p. 238–240 °C (EtOH/CHCl_3_); Mol. Formula (Mol. Wt.): C_18_H_21_Cl_2_N_3_S (382.35). ^1^H NMR (DMSO-*d*_6_, 700.17 MHz): δ 1.61–1.65 (m, 6H, Adamantane-H), 2.05 (s, 3H, Adamantane-H), 2.21 (s, 6H, Adamantane-H), 7.38–7.41 (m, 1H, Ar-H), 7.54–7.57 (m, 2H, Ar-H & NH), 8.37 (s, 1H, CH=N), 11.69 (s, 1H, NH). ^13^C NMR (DMSO-*d*_6_, 176.08 MHz): δ 29.43, 36.28, 41.32, 53.41 (Adamantane-C), 129.5, 130.19, 131.26, 134.16 (Ar-C), 136.07 (CH=N), 175.35 (C=S). ESI-MS, *m*/*z*: 380.4 [M − H]^−^, 382.4 [M + 2 − H]^−^.

*4-(Adamantan-1-yl)-1-[(benzo[d]*[1,3]*dioxol-6-yl)methylene]-3-thiosemicarbazide***4g**: Yield 82%; m.p. 184–186 °C (EtOH); Mol. Formula (Mol. Wt.): C_19_H_23_N_3_O_2_S (357.47). ^1^H NMR (DMSO-*d*_6_, 700.17 MHz): δ 1.63–1.66 (s, 6H, Adamantane-H), 2.04–2.06 (m, 3H, Adamantane-H), 2.18 (s, 3H, Adamantane-H), 2.26 (s, 3H, Adamantane-H), 6.12 (s, 2H, OCH_2_O), 6.88–6.95 (m, 2H, Ar-H), 7.19 (d, 1H, Ar-H, *J* = 7.0 Hz), 7.57 (s, 1H, NH), 8.40 (s, 1H, CH=N), 11.46 (s, 1H, NH). ^13^C NMR (DMSO-*d*_6_, 176.08 MHz): δ 29.46, 36.43, 41.32, 53.42 (Adamantane-C), 102.03 (OCH_2_O), 109.72, 116.93, 119.33, 122.38, 146.32, 148.24 (Ar-C), 136.08 (CH=N), 174.98 (C=S). ESI-MS, *m*/*z*: 356.3 [M − H]^−^.

### 3.4. N-(Adamantan-1-yl)-4-methylpiperazine-1-carbothioamide ***5***

A mixture of 1-adamantyl isothiocyanate **2** (387 mg, 2 mmol) and 1-methylpiperazines (200 mg, 2.0 mmol), in ethanol (15 mL), was heated under reflux for two hours. On cooling, the precipitated crude product was filtered, washed with cold ethanol, dried, and crystallized from *n*-hexane to yield 470 mg (80%) as transparent needle crystals; m.p. 184–186 °C; Mol. Formula (Mol. Wt.): C_16_H_27_N_3_S (293.47). ^1^H NMR (DMSO-*d*_6_, 700.17 MHz): δ 1.62 (s, 6H, Adamantane-H), 2.03 (s, 3H, Adamantane-H), 2.18 (s, 3H, CH_3_), 2.24 (s, 6H, Adamantane-H), 2.27 (t, 4H, Piperazine-H, *J* = 4.9 Hz), 3.67 (t, 4H, Piperazine-H, *J* = 4.9 Hz), 6.54 (s, 1H, NH). ^13^C NMR (DMSO-*d*_6_, 176.08 MHz): δ 29.56, 36.57, 41.39, 54.20 (Adamantane-C), 45.92 (CH_3_), 47.61, 54.85 (Piperazine-C), 180.82 (C=S). ESI-MS, *m*/*z*: 294.0 [M + H]^+^.

### 3.5. 4-Arylmethyl N′-(adamantan-1-yl)piperidine-1-carbothioimidates ***7a–c***

The appropriate arylmethyl bromide (2 mmol) and anhydrous potassium carbonate (276 mg, 2 mmol) were added to a stirred solution of *N*-(adamantan-1-yl)piperidine-1-carbothioamide **6** (557 mg, 2 mmol), in *N*,*N*-dimethylformamide (10 mL), and the mixture was stirred for 24 h at room temperature. Cold water (20 mL) was then added and the precipitated crude products were filtered, washed with water, dried, and crystallized from ethanol to yield the target compounds **7a–c** as transparent block crystals.

*4-Benzyl N′-(adamantan-1-yl)piperidine-1-carbothioimidate***7a**: Yield 78%; m.p. 79–80 °C; Mol. Formula (Mol. Wt.): C_23_H_32_N_2_S (368.23). ^1^H NMR (DMSO-*d*_6_, 700.17 MHz): δ 1.55–1.75 (m, 18H, Adamantane-H & Piperidine-H), 1.92 (s, 3H, Adamantane-H), 3.14–3.16 (m, 4H, Piperidine-H), 3.97 (s, 2H, Benzylic CH_2_), 7.29–7.30 (m, 5H, Ar-H). ^13^C NMR (DMSO-*d*_6_, 176.08 MHz): δ 24.0, 25.81, 50.16 (Piperidine-C), 29.74, 36.58, 43.17, 54.20 (Adamantane-C), 37.0 (Benzylic CH_2_), 127.36, 128.78, 129.17, 138.86 (Ar-C), 151.0 (C=N). ESI-MS, *m*/*z*: 369.1 [M + H]^+^.

*4-Bromobenzyl N′-(adamantan-1-yl)piperidine-1-carbothioimidate***7b**: Yield 86%; m.p. 96–98 °C; Mol. Formula (Mol. Wt.): C_23_H_31_BrN_2_S (447.47). ^1^H NMR (CDCl_3_, 700.17 MHz): δ 1.63–2.19 (m, 21H, Adamantane-H & Piperidine-H), 3.91-3.93 (m, 4H, Piperidine-H), 4.13 (s, 2H, Benzylic CH_2_), 7.19 (d, 2H, Ar-H, *J* = 7.0 Hz), 7.52 (d, 2H, Ar-H, *J* =7 Hz). ^13^C NMR (CDCl_3_, 176.08 MHz): δ 23.54, 26.04, 53.77 (Piperidine-C), 29.63, 35.61, 42.42, 59.14 (Adamantane-C), 39.70 (Benzylic CH_2_), 122.93, 130.52, 132.51, 133.23 (Ar-C), 167.73 (C=N). ESI-MS, *m*/*z*: 449.1 [M + 2 + H]^+^, 447.1 [M + H]^+^.

*4-Nitrobenzyl N′-(adamantan-1-yl)piperidine-1-carbothioimidate***7c***:* Yield 94%; m.p. 116–118 °C; Mol. Formula (Mol. Wt.): C_23_H_31_N_3_O_2_S (413.58). ^1^H NMR (DMSO-*d*_6_, 700.17 MHz): δ 1.53–1.57 (m, 12H, Adamantane-H & Piperidine-H), 1.71 (s, 6H, Adamantane-H), 1.91 (s, 3H, Adamantane-H), 3.11–3.13 (m, 4H, Piperidine-H), 4.10 (s, 2H, Benzylic CH_2_), 7.54 (d, 2H, Ar-H, *J* = 7.0 Hz), 8.19 (d, 2H, Ar-H, *J* = 7.0 Hz). ^13^C NMR (DMSO-*d*_6_, 176.08 MHz): δ 25.04, 25.70, 50.25 (Piperidine-C), 29.70, 36.50, 43.16, 54.29 (Adamantane-C), 37.02 (Benzylic CH_2_), 123.94, 130.47, 146.74, 147.31 (Ar-C), 150.0 (C=N). ESI-MS, *m*/*z*: 414.1 [M + H]^+^.

### 3.6. 1,3-Bis(adamantan-1-yl)thiourea ***8***

A mixture of 1-adamantylamine **2** (1.51 g, 0.01 mol) and 1-adamantyl isothiocyanate **3** (1.93 g, 0.01 mol), in ethanol (10 mL), was heated under reflux for four hours. On cooling, the precipitated crude product was filtered, washed with cold ethanol, dried, and crystallized from ethanol to yield 2.58 g (75%) of the title compound **8** as white amorphous powder; m.p. 161–163 °C; Mol. Formula (Mol. Wt.): C_21_H_32_N_2_S (344.56). ^1^H NMR (DMSO-*d*_6_, 700.17 MHz): δ 1.61 (s, 12H, Adamantane-H), 2.01 (s, 6H, Adamantane-H), 2.15 (s, 12H, Adamantane-H), 6.84 (s, 2H, NH). ^13^C NMR (DMSO-*d*_6_, 176.08 MHz): δ 29.48, 36.53, 41.70, 53.12 (Adamantane-C), 179.85 (C=S). ESI-MS, *m*/*z*: 345.0 [M + H]^+^.

### 3.7. 3-(Adamantan-1-yl)-2-(adamantan-1-ylimino)lthiazolidin-4-one ***9***

A mixture of 1,3-bis(adamantan-1-yl)thiourea **8** (690 mg, 2.0 mmol), chloroacetic acid (190 mg, 2.0 mmol), and anhydrous sodium acetate (165 mg, 2.0 mmol), in ethanol (1.0 mL), was irradiated in microwave oven for 10 min at a maximum power of 700 W. Cold water (20 mL) was then added to the mixture and the precipitated crude products were filtered, washed with water, dried, and crystallized from ethanol to yield 554 mg (72%) of the title compound **9** as white amorphous powder; m.p. 256–258 °C; Mol. Formula (Mol. Wt.): C_23_H_32_N_2_OS (384.58). ^1^H NMR (CDCl_3_, 700.17 MHz): δ 1.60–1.70 (m, 12H, Adamantane-H), 1.82 (s, 3H, Adamantane-H), 1.97–2.11 (m, 15H, Adamantane-H), 3.87 (s, 2H, SCH_2_). ^13^C NMR (CDCl_3_, 176.08 MHz): δ 29.58, 29.86, 36.06, 36.48, 42.57, 44.80, 50.90, 55.0 (Adamantane-C), 139.50 (C=N), 158.0 (C=O). ESI-MS, *m*/*z*: 385.1 [M + H]^+^.

### 3.8. 3-(Adamantan-1-yl)-2-(adamantan-1-ylimino)-5-arylidenethiazolidin-4-ones ***11a,b***

A mixture of 1,3-bis(adamantan-1-yl)thiourea **8** (345 mg, 1.0 mmol), chloroacetic acid (95 mg, 1.0 mmol), the appropriate aromatic aldehyde (1.0 mmol), and anhydrous sodium acetate (82 mg, 1.0 mmol), in ethanol (1.0 mL), was irradiated in a microwave oven for 10 min at a maximum power of 700 W. Cold water (20 mL) was then added to the mixture and the precipitated crude products were filtered, washed with water, dried, and crystallized from ethanol to yield the target compound **11a,b** as white amorphous powder.

*3-(Adamantan-1-yl)-2-(adamantan-1-ylimino)-5-(4-chlorobenzylidene)-thiazolidin-4-one***11a**: Yield 45%; m.p. 247–249 °C; Mol. Formula (Mol. Wt.): C_30_H_35_ClN_2_OS (507.13). ^1^H NMR (CDCl_3_, 700.17 MHz): δ 1.68–1.7 (m, 3H, Adamantane-H), 1.76–1.77 (m, 9H, Adamantane-H), 2.04 (s, 6H, Adamantane-H), 2.16–2.18 (m, 6H, Adamantane-H), 2.70 (s, 6H, Adamantane-H), 7.43 (s, 1H, CH=C), 7.44–7.46 (m, 4H, Ar-H). ^13^C NMR (CDCl_3_, 176.08 MHz): δ 29.69, 30.49, 36.42, 36.45, 39.52, 42.22, 56.43, 65.69 (Adamantane-C), 124.63 (C-5), 124.98, 129.1, 130.81, 135.0 (Ar-C), 136 (Ethylene-C), 137.59 (C=N), 166.54 (C=O). ESI-MS, *m*/*z*: 509.1 [M + 2 + H]^+^, 507.1 [M + H]^+^.

*3-(Adamantan-1-yl)-2-(adamantan-1-ylimino)-5-(4-nitrobenzylidene)-thiazolidin-4-one***11b**: Yield 28%; m.p. 288–290 °C; Mol. Formula (Mol. Wt.): C_30_H_35_N_3_O_3_S (517.68). ^1^H NMR (DMSO-*d*_6_, 500.15 MHz): δ 1.68–1.70 (m, 15H, Adamantane-H), 2.11–2.17 (m, 15H, Adamantane-H), 7.70 (s, 1H, CH=C), 7.81 (d, 2H, Ar-H, *J* = 7.0 Hz), 8.36 (d, 2H, Ar-H, *J* = 7.0 Hz). ^13^C NMR (DMSO-*d*_6_, 176.08 MHz): δ 29.33, 36.04, 40.64, 41.03, 52.45, 56.47 (Adamantane-C), 124.77 (C-5), 126.49, 130.73, 141.37, 147.39 (Ar-C), 134.87 (C-ethylene), 170.68 (C=N), 180.12 (C=O). ESI-MS, *m*/*z*: 517.0 [M + H]^+^.

## 4. Conclusions

A series of adamantane-linked thiosemicarbazones (**4a–g**), isothioureas (**7a–c**), and thiazolidin-4-ones (**9**, **11a**, **11b**) was prepared and characterized, and their in vitro antimicrobial and anti-proliferative activities were evaluated. The adamantyl isothiourea derivatives **7a–c** displayed strong broad-spectrum antibacterial activity (MIC, 0.5–32 μg/mL) and the thiosemicarbazone derivatives **4a** and **4g** showed marked antifungal activity against *Candida albicans*. The anti-proliferative activity assessment of **4a**, **4d**, **4f**, **4g**, **7a**, **7b**, **7c**, **9**, and **11a** against the human tumor cell lines HL-60, HT-29, and MCF7 revealed that the isothiourea derivatives **7a–c** are highly active, with IC_50_ < 10 μM against the tested cell lines, and the thiosemicarbazone derivatives showed moderate activity, with IC_50_ values >10–50 μM. It could be concluded that adamantane-linked isothioureas (**7a–c**) and, to a lesser extent, the thiosemicarbazones (**4a–g**), are considered to be good candidates as newer antibacterial, antifungal, and anticancer agents. The biological screening results are considered as preliminary and further investigations including experimental and molecular docking studies for the exploration of the mechanism of their biological activity are required for optimization of their chemotherapeutic activities.

## Figures and Tables

**Table 1:**
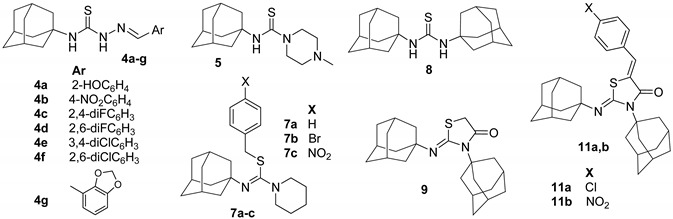
In vitro antimicrobial activity of compounds **4a–g**, **5**, **7a–c**, **8**, **9**, **11a**, and **11b** (200 μg/8 mm disc); the broad-spectrum antibacterial drugs Gentamicin sulfate, Ampicillin trihydrate, and the antifungal drug Clotrimazole (100 μg/8 mm disc) against *Staphylococcus aureus* American type culture collection (ATCC) 6571 (SA), *Bacillus subtilis* ATCC 5256 (BS), *Micrococcus luteus* ATCC 27141 (ML), *Escherichia coli* ATCC 8726 (EC), *Pseudomonas aeruginosa* ATCC 27853 (PA), and the yeast-like pathogenic fungus *Candida albicans* MTCC 227 (CA).

Comp. No.	Clog *P*	Diameter of Growth Inhibition Zone (mm) ^a^					
		SA	BS	ML	EC	PA	CA
**4a**	4.605	24 (4) ^b^	27 (2) ^b^	22 (2) ^b^	18 (8) ^b^	17	19 (8) ^b^
**4b**	4.273	16	17	14	11	-	11
**4c**	4.816	19 (8) ^b^	22 (4) ^b^	20 (8) ^b^	16	12	12
**4d**	4.816	27 (1) ^b^	29 (0.5) ^b^	23 (2) ^b^	22 (2) ^b^	19 (8) ^b^	12
**4e**	5.836	20 (4) ^b^	21 (4) ^b^	18 (32) ^b^	13	11	-
**4f**	5.956	29 (0.5) ^b^	31 (0.5) ^b^	28 (1) ^b^	23 (2) ^b^	18 (16) ^b^	16
**4g**	4.840	22 (2) ^b^	22 (2) ^b^	19 (8) ^b^	14	12	18 (32) ^b^
**5**	3.793	14	16	11	-	-	-
**7a**	6.984	22 (1) ^b^	26 (1) ^b^	19 (4) ^b^	20 (16) ^b^	17	12
**7b**	7.847	29 (0.5) ^b^	32 (0.5) ^b^	21 (1) ^b^	22 (2) ^b^	18 (8) ^b^	11
**7c**	6.727	24 (1) ^b^	22 (0.5) ^b^	18 (16) ^b^	23 (4) ^b^	18 (32) ^b^	12
**8**	5.050	11	13	-	-	-	-
**9**	5.499	-	-	-	-	-	-
**11a**	8.445	13	12	12	-	-	-
**11b**	7.475	11	12	-	-	-	-
**Gentamicin sulfate**		27 (1) ^b^	26 (2) ^b^	20 (2) ^b^	22 (0.5) ^b^	21 (0.5) ^b^	NT
**Ampicillin trihydrate**		22 (2) ^b^	23 (1) ^b^	20 (2) ^b^	16 (8) ^b^	16 (8) ^b^	NT
**Clotrimazole**		NT	NT	NT	NT	NT	21 (4) ^b^

^a^ (-): inactive (inhibition zone < 10 mm), ^b^ Figures shown in parentheses represent the minimal inhibitory concentration (MIC) values (μg/mL), NT: not tested. SA, *Staphylococcus aureus*; BS, *Bacillus subtilis*; ML, *Micrococcus luteus*; EC, *Escherichia coli*; PA, *Pseudomonas aeruginosa*; CA, *Candida albicans*.

**Table 2:** In vitro anti-proliferative activity of the tested compounds **4a**, **4d**, **4f**, **4g**, **7a**, **7b**, **7c**, **9**, **11a**, and Doxorubicin expressed as IC_50_ values against HL-60 (human promyelocytic leukemia cell line), HT-29 (human colorectal cancer cell line), and MCF7 (human breast cancer cell line).

Comp. No.	IC_50_ (µM) ^a^		
	HL-60	HT-29	MCF7
**4a**	>100	75.60 ± 0.77	92.67 ± 2.70
**4d**	32.65 ± 1.52	25.46 ± 1.22	22.40 ± 1.20
**4f**	24.98 ± 2.02	47.50 ± 1.27	32.75 ± 2.90
**4g**	24.24 ± 1.33	18.99 ± 1.02	24.02 ± 0.88
**7a**	6.62 ± 0.52	4.25 ± 0.08	1.55 ± 0.04
**7b**	5.30 ± 0.99	3.67 ± 1.02	0.95 ± 0.09
**7c**	8.43 ± 1.01	2.68 ± 0.32	0.46 ± 0.33
**9**	>100	>100	>100
**11a**	>100	>100	>100
**Doxorubicin**	1.05 ± 0.12	0.32 ± 0.02	0.11 ± 0.10

^a^ IC_50_ values presented as the mean ± SD of three separate determinations.

## Supplementary Materials

The microanalytical data (C, H, N and S) and the experimental details of the determination of in vitro antimicrobial activity, in vitro anti-proliferative activity can be found at https://www.mdpi.com/1420-3049/24/23/4308/s1.
