# Supplementary material for: Synthesis, Antimicrobial, and Anti-Proliferative Activities of Novel 4-(Adamantan-1-yl)-1-arylidene-3-thiosemicarbazides, 4-Arylmethyl N′-(Adamantan-1-yl)piperidine-1-carbothioimidates, and Related Derivatives
Source: Molecules. 2019 Nov 26;24(23):4308. doi: 10.3390/molecules24234308 (PMC6930481; doi:10.3390/molecules24234308)
Supplement: Supplementary file 1 [file molecules-24-04308-s001.pdf]

Supplementary Materials

Synthesis, Antimicrobial and Anti-proliferative Activities of Novel 4-(Adamantan-1-yl)-1-arylidene-3-thiosemicarbazides, 4-Arylmethyl N'-(adamantan-1-yl)piperidine-1-carbothioimidates and Related Derivatives

Table S1. Molecular formulae, molecular weights and elemental analyses data of compounds **4a-g**, **5**, **7a-c**, **8**, **9**, **11a** and **11b**.

Comp. No.	Mol. Formula (Mol. Wt.)	Analysis: % Calcd. (Found)			
		C	H	N	S
**4a**	$C_{18}H_{23}N_3OS$ (329.46)	65.62 (65.44)	7.04 (7.12)	12.75 (12.65)	9.73 (9.68)
**4b**	$C_{18}H_{22}N_4O_2S$ (358.46)	60.31 (65.11)	6.19 (6.22)	15.63 (15.62)	8.95 (8.88)
**4c**	$C_{18}H_{21}F_2N_3S$ (349.44)	61.87 (61.80)	6.06 (6.24)	12.02 (12.0)	9.18 (9.21)
**4d**	$C_{18}H_{21}F_2N_3S$ (349.44)	61.87 (61.75)	6.06 (6.11)	12.02 (11.98)	9.18 (9.16)
**4e**	$C_{18}H_{21}Cl_2N_3S$ (382.35)	56.54 (56.62)	5.54 (5.60)	10.99 (11.03)	8.39 (8.36)
**4f**	$C_{18}H_{21}Cl_2N_3S$ (382.35)	56.54 (56.40)	5.54 (5.50)	10.99 (10.89)	8.39 (8.41)
**4g**	$C_{19}H_{23}N_3O_2S$ (357.47)	63.84 (63.58)	6.49 (6.55)	11.75 (11.68)	8.97 (9.01)
**5**	$C_{16}H_{27}N_3S$ (293.47)	65.48 (65.45)	9.27 (9.40)	14.32 (14.18)	10.93 (10.88)
**7a**	$C_{23}H_{32}N_2S$ (368.23)	74.95 (74.77)	8.75 (8.90)	7.60 (7.44)	8.70 (8.70)
**7b**	$C_{23}H_{31}BrN_2S$ (447.14)	61.73 (61.70)	6.98 (7.11)	6.26 (6.32)	7.17 (7.13)
**7c**	$C_{23}H_{31}N_3O_2S$ (413.58)	66.79 (66.82)	7.56 (7.62)	10.16 (10.22)	7.75 (7.68)
**8**	$C_{21}H_{32}N_2S$ (344.56)	73.20 (72.96)	9.36 (9.44)	8.13 (8.12)	9.31 (9.36)
**9**	$C_{23}H_{32}N_2OS$ (384.58)	71.83 (71.85)	8.39 (8.50)	7.28 (7.25)	8.34 (8.41)
**11a**	$C_{30}H_{35}ClN_2OS$ (507.13)	71.05 (69.85)	6.96 (7.10)	5.52 (5.52)	6.32 (6.22)
**11b**	$C_{30}H_{35}N_3O_3S$ (517.68)	69.60 (69.33)	6.81 (6.80)	8.12 (8.10)	6.19 (6.20)

Determination of in vitro antimicrobial activity (agar disc diffusion method)

Sterile filter paper discs (8 mm diameter) were moistened with the compound solution in dimethyl sulfoxide of specific concentration (200 μg/disc), and the antibacterial drugs, Gentamicin sulphate and Ampicillin trihydrate (100 μg/disc) and the antifungal drug Clotrimazole (100 μg/disc), were carefully placed on agar culture plates that had been previously inoculated separately with the microorganisms. The plates were incubated at 37 °C, and the diameters of the growth inhibition zones were measured after 24 hours for bacteria and 48 hours for *C. albicans*.

Determination of the minimal inhibitory concentration (MIC)

Compounds **4a**, **4c**, **4d**, **4e**, **4f**, **4g**, **7a**, **7b**, **7c**, Gentamicin sulfate, Ampicillin trihydrate, and Clotrimazole were dissolved in dimethyl sulphoxide at a concentration of 128 μg/mL. The two-fold dilutions of the solution were prepared (128, 64, 32, …, 0.25 μg/mL). Suspensions of the microorganisms at concentrations of 106 colony-forming units per mL were inoculated in the corresponding wells. The plates were then incubated at 36°C for 24 hours. The MIC values were determined as the lowest concentrations that completely inhibited visible growth of the microorganism as detected by the unaided eye.

Determination of in vitro anti-proliferative activity

The *in vitro* anti-proliferative activity screening of compounds **4a**, **4d**, **4f**, **4g**, **7a**, **7b**, **7c**, **9** and **11a** was measured against three human tumor cell lines namely; HL-60 (human promyelocytic leukemia cell line), HT-29 (human colorectal cancer cell line) and MCF7 (human breast cancer cell line) using the standard MTT assay. HL-60, HT-29 and MCF7 cells (3000 cells per well) were cultured and seeded into 96-well plates and the plates were incubated for 24 hours. The cells were then treated with compounds **4a**, **4d**, **4f**, **4g**, **7a**, **7b**, **7c**, **9**, **11a** and Doxorubicin at different concentrations in DMSO (0.1 μM to 100 μM) at 37 °C in an atmosphere of 5% CO_2 for 48 hours. Freshly prepared MTT was added to each well at a terminal concentration of 5 μg/mL and incubated with cells at 37 °C for 4 hours. The formazan crystals were dissolved in 100 μL of DMSO in each well, and the absorbency at 492 nm (for absorbance of MTT formazan) and 630 nm (for the reference wavelength) was measured with an enzyme linked immunosorbent assay (ELISA) reader (ChroMate-4300, FL, USA). All compounds were tested three times in each of the cell lines. The IC_{50} values were calculated according to the equation for Boltzmann sigmoidal concentration response curve using the nonlinear regression fitting models (Graph Pad, Prism Version 5). The results reported are means of three separate experiments. Statistical differences were analysed according to one way ANOVA test wherein the differences were considered to be significant at $p < 0.05$.
